# What works? A framework analysis of breastfeeding support provider perspectives in a low-breastfeeding region with baby friendly accreditation

**DOI:** 10.1186/s13006-026-00881-2

**Published:** 2026-08-18

**Authors:** Mary Jo Chesnel, Maria Healy, Jenny McNeill

**Affiliations:** School of Nursing and Midwifery, Queen’s University Belfast, Belfast, Northern UK

**Keywords:** Breast feeding, Midwifery, Health services, Breastfeeding support, Peer supporters, Breastfeeding education

## Abstract

**Background:**

Evidence-based breastfeeding support interventions can improve breastfeeding rates. However, in Northern Ireland breastfeeding rates remain amongst the lowest worldwide despite 100% UNICEF BFI accreditation of maternity units, and the availability of community and private breastfeeding support. There is limited understanding of the provider perspective on factors that influence their ability to provide effective support in such contexts. This study aimed to explore the provider perspective of supporting women to breastfeed across a range of role-types in a context with low breastfeeding rates, yet high rates of UNICEF BFI accreditation across maternity and family settings.

**Methods:**

Semi-structured interviews were conducted with trained breastfeeding support providers employed in healthcare roles (*n* = 12) and lay trained providers (*n* = 9). A thematic framework analysis was undertaken.

**Results:**

Three key themes were developed from the dataset: *Role development through active engagement*,* Positivity: Breastfeeding support as meaningful work*, and *Negativity: Precarity in quality support provision*. Findings suggest a range of positive and negative factors underpinning support provision. Positive factors were most evident when providers were regularly exposed to positive breastfeeding support encounters with women, and where breastfeeding was normalised, understood and valued within healthcare systems and by individuals.

**Conclusions:**

Breastfeeding support providers develop confidence and skill through experiential learning and collaboration with others who value breastfeeding. This can be undermined by limited exposure to breastfeeding in low-breastfeeding contexts, and a lack of support for providers themselves in practice despite a system’s BFI accreditation status. Providers can therefore struggle to fully enact their breastfeeding support role despite UNICEF BFI accreditation. What works best for providers to support breastfeeding women is continued exposure to breastfeeding dyads, ongoing peer learning from other providers who value and understand breastfeeding practices, and strong team cohesion across professional and lay-trained roles.

**Supplementary Information:**

The online version contains supplementary material available at https://doi.org/10.1186/s13006-026-00881-2.

## Background

Breastfeeding has an unparallelled positive impact on maternal and child physical and psychological health, and the environment [1–4]. Despite attention in recent decades to improving rates as a public health issue, global rates remain low and there is a need to upscale breastfeeding protection, promotion and support to meet WHO targets for breastfeeding exclusivity and duration [1, 5]. Breastfeeding support - defined here as informational, practical and emotional support provided by trained providers to women in acute and community settings [6] is an evidence-based intervention which can increase breastfeeding rates [7–9]. It is increasingly promoted at policy-level in areas where breastfeeding is no longer the cultural norm [10, 11]. Yet, breastfeeding rates may not always rise following such interventions, particularly in well-resourced settings [8, 12] and women are not always satisfied with the support they receive [13, 14]. Insufficient and ineffective support provision is a leading cause of early unplanned breastfeeding cessation [7, 15]. In low breastfeeding settings, cultural influence and a lack of social support for breastfeeding also negatively impact women’s breastfeeding intentions and practices [16–18]. There is therefore evidence that breastfeeding support services in such contexts could be improved [13, 19].

Many countries have implemented UNICEF BFI accreditation [10] of healthcare services as an intervention to improve breastfeeding support. This framework of evidence-based strategies and standards helps promote, protect and support breastfeeding in healthcare settings. In Northern Ireland, 100% of Health and Social Care Trust (HSCT) maternity and health visiting settings are UNICEF BFI accredited. HSC Trusts are publicly funded integrated health and social care providers responsible for NI regional service provision within the wider UK National Health Service. In the UK, *health visiting* is a universal early years service for families with young children (0–5 years) delivered by registered nurses and midwives with a specialist post-registration public health qualification. The health visitor provides an infrastructure of support for infants, children and families across physical and mental health, child development, social needs and safeguarding through home visits and community-based early intervention and care. In addition to recommending UNICEF BFI accreditation of services, the regional breastfeeding strategy promotes breastfeeding support in community and social settings [20]. However, despite incremental increases following the implementation of the regional breastfeeding strategy [20] Northern Ireland breastfeeding rates are consistently lowest in comparison with other well-resourced countries. For example, in 2022 50.8% women were breastfeeding on discharge home after birth, with rates dropping to 34.6% of 6–8 week old infants getting any breastmilk [21] compared to 52.7% infants in England getting any breastmilk at 6–8 weeks in the year 2023/24 [22], and 71% of infants receiving breastmilk at four months of age in Australia in 2022 [23].

Women in Northern Ireland are cared for by UNICEF BFI trained providers such as healthcare staff in the intranatal and postnatal period, and women have access to proactive and reactive breastfeeding support from a range of other trained providers in postpartum weeks and months. Yet despite 62.2% women initiating breastfeeding, only 34.6% women are breastfeeding at six weeks [21]. Since all women would have been cared for by midwives and health visitors trained in breastfeeding support, and possibly by other trained providers such as Health and Social Care Trust (HSCT) peer supporters, trained community breastfeeding support providers, International Board Certified Lactation Consultants (IBCLCs) and General Practitioners, the almost 50% drop in rates from birth to six weeks suggests a need for improvement in support provision, even within UNICEF BFI accredited settings.

The difficulty in supporting women to breastfeed in contemporary societies where breastfeeding is no longer the cultural norm and where commercial interest in infant feeding is increasing is well established [24]. Breastfeeding is both a personal and social experience and so consideration of context is crucial for effective implementation of interventions [25] such as UNICEF BFI. Northern Ireland scored 10/10 for the indicator titled ‘Counselling services for pregnant and breastfeeding mothers’ in the most recent World Breastfeeding Trends Initiative (WBTI) UK report [26], an initiative which evaluates the provision of breastfeeding support globally. This maximum score was attributed to Northern Ireland’s 100% BFI accreditation status. UNICEF BFI accreditation of maternity units therefore serves a proxy indicator of high-quality support provision [26]. However, such proxy measures fail to capture the detail of everyday practice or the extent to which standards are sustained once staff are trained and support staff are appointed. Indeed, there is some evidence of implementation slide. In comparison to the 2016 WBTI report, Northern Ireland scored lower in the 2024 report on indicators relating to monitoring and evaluation of breastfeeding, and also those relating to enforcement of regulations relating to the International Code of Marketing of Breastmilk substitutes [26]. A comprehensive understanding of how best to provide evidence-based breastfeeding support services in low breastfeeding contexts is therefore still needed, notwithstanding BFI accreditation. For example, in Northern Ireland with its 100% BFI accreditation of maternity and family services, breastfeeding rates are rising incrementally but remain very low, with a sharp drop in rates in the early days and weeks [21], which raises questions about the contextual factors influencing support provision. There is still little understanding of what works well to overcome challenges that persist in supporting women effectively after BFI accreditation. The perspectives of both trained professional and lay support providers on the factors that influence how they support breastfeeding women after BFI accreditation is underexplored in the literature. This study aimed to address this knowledge gap to direct improvements in service delivery and design in such contexts, and generate fresh insights into what works well in breastfeeding support provision.

## Methods

### Study aim

This research aimed to explore the perspectives of breastfeeding support providers across role-types, focusing on factors which influence breastfeeding support provision in a society with persistently low breastfeeding rates despite high levels of BFI accreditation.

### Study setting

The study was Northern Ireland wide and included representation of providers in a range of UNICEF BFI accredited settings. All women in Northern Ireland receive face-to-face postnatal support from midwives, maternity support workers and, later, health visitors all of whom are trained under UNICEF BFI. The study explored support provision across usual postnatal settings including maternity units, women’s homes, community clinical settings and Sure Start settings. *Sure Start* is a publicly funded early intervention programme supporting children pre-birth to school age, delivered in community settings in areas of social disadvantage. Programmes such as breastfeeding support groups are delivered through partnerships between Sure Start and HSCTs, community organisations and early years providers. Additional support for women includes Trust peer supporters offering a telephone and text-based service, and there are specialist breastfeeding health professionals in each Trust, who may be qualified IBCLCs. Women experiencing breastfeeding challenges may also contact General Practitioner medical doctors (GP), and independent lactation services such as IBCLCs in private practice.

### Study design

This paper reports on one aspect of a research project exploring multiple aspects of breastfeeding support provision in Northern Ireland. The wider study includes a metasynthesis investigating experiences which influence trained provider practice and a hermeneutic phenomenology of women’s experiences of breastfeeding support which will be reported separately. The literature review has been published [27] and its findings informed the development of the topic guide used in this study. This paper, however, focuses on the study of provider perspectives on breastfeeding support provision which involved a descriptive qualitative inquiry using a thematic framework analysis [28]. The qualitative design facilitated an exploration of participants’ perspectives, with the analytical method involving the development of a framework matrix that facilitated identification of similarities and differences within the dataset. This enabled comparison across cases in the matrix, allowing exploration of trends in perspectives on the topic of breastfeeding support provision across differing support roles, which aligns with the aim of the research.

### Participants and recruitment

The sample comprised individuals trained to support breastfeeding women who practice in two distinct roles: those supporting women as part of a generic healthcare role, and those not employed in a healthcare role. See S1 for eligibility criteria.

A Project Advisory Group (PAG) was set up comprising representatives from Health and Social Care Trusts (HSCTs), midwifery education and research, the Public Health Agency and breastfeeding community advocacy and support organisations which included breastfeeding women. The PAG distributed recruitment flyers via their networks to midwives, health visitors and trained maternity support workers using purposive and snowball techniques. Recruitment also centred on two HSCTs providing Maternity and Women and Child Health Services in acute and community settings. HSCT trained breastfeeding supporters who are not employed in healthcare roles, but volunteer, were recruited via social media platforms.

### Ethics

Prior to commencing the study ethical and governance approval were obtained from the Research Ethics Committee (IRAS ID 279407) and HSCT Governance. Participants freely gave informed consent, anonymity was preserved, there was no inducement to participate, and data security was in line with University Research data management policy.

### Data collection

A demographic form, participant information sheet and a digital consent form were sent to each participant and consent reconfirmed verbally before data collection. Online one-to-one semi-structured interviews were conducted by the first author MJC using a topic guide (S2) developed from a related qualitative evidence synthesis [27] and informed by the PAG. Interviews lasted between 36 and 96 min and were audio-recorded and transcribed digitally, with identifying information removed. The data was coded to a pre-defined thematic structure based on priori knowledge [29] namely the qualitative evidence synthesis. Sample size was guided by the study aim, design, and available resources, not data saturation. This was not a prerequisite because Framework Analysis involves using a thematic structure developed from a priori knowledge, therefore the concept of developing new themes until saturation does not apply. The final sample size was sufficient for a framework analysis because several participants represented each role type, enabling comparison of perspectives across a range of healthcare and non-healthcare employee breastfeeding support roles.

### Data analysis

A thematic framework analysis was conducted to identify commonalities and disparities in the dataset, using the framework matrices section of QSR NVivo 12 qualitative software. Analysis was undertaken with input from the three authors MJC, MH and JM, following the seven stages of thematic framework analysis [28] . Following open team coding of a subset of transcripts chosen for rich data across the range of roles, we developed a codebook and from this, agreed a thematic framework through consensus between authors. The framework was then imposed on the remainder of the dataset. The resultant matrix output was interpreted by the authors to elicit patterns of meaning about breastfeeding support provision. During analysis authors agreed that the factors underpinning breastfeeding support provision were closely linked to how breastfeeding was valued at societal, systemic and individual levels. The valuing or undervaluing of breastfeeding was reflected in how breastfeeding was normalised in society, the availability of adequate staffing and resources for breastfeeding support within the healthcare system, and whether trained support providers actively sought to engage with breastfeeding women.

### Reflexivity

All three researchers are registered midwives and midwifery educators with individual experiences of providing breastfeeding support and receiving breastfeeding support as mothers. Our familiarity with the topic has been considered. Strategies to mitigate against undue researcher influence were employed including shared critical examination of interpretations within the team, and MJC regularly updated a research diary with reflections to enable critique of undue influence on the research process.

### Findings

Participants were recruited between December 2020 and August 2021. This analysis reports on 21 participants comprising healthcare employees HCR *n* = 12, and non-healthcare NHCR employees *n* = 9. The sample reflects an experienced UNICEF BFI trained support workforce in a range of roles, supporting women in maternity units, community settings including health visiting, Sure Start and Continuity of Midwifery Care teams, and remotely via telephone support. Midwives are the largest represented healthcare employee group alongside a small number of health visitors and a maternity support worker. One midwife and one health visitor were qualified IBCLCs. Peer supporters make up the entirety of the non-healthcare employee group. At the time of data collection, no trained providers reported working within a dedicated multisector breastfeeding team. Participants’ ages ranged from 31 to over 61 years, with between 10 months and 35 years’ experience of supporting breastfeeding women in face-to-face and telephone interactions. Sample characteristics are presented in Table 1.

**Table 1:** Participant demographics

Characteristic	*n*
*Age in years*	
31–40	9
41–50	6
51–60	4
61+	2
*Role*	
Midwife (Healthcare employee role HCR)	9
Health visitor (HCR)	2
Maternity support worker (HCR)	1
Peer supporter (Non-healthcare employee role NHCR)	9
*Breastfeeding support setting*	
Acute maternity unit: face-to-face	6
Community: face-to-face	2
Community and Sure Start community: face-to-face	3
Community - Continuity of Care model: face-to-face	1
HSCT (Health and Social Care Trust) Peer support service: telephone	8
HSCT Peer support service: telephone, also Sure Start face-to-face	1
*Duration of breastfeeding support experience*	
6 months – 1 yr	2
1–5 yrs	6
5-10yrs	3
10-15yrs	3
15-20yrs	2
20-25yrs	2
25-30yrs	1
30-35yrs	2

*All peer supporters were trained in UNICEF BFI accredited HSCT maternity units

**Fig. 1:**
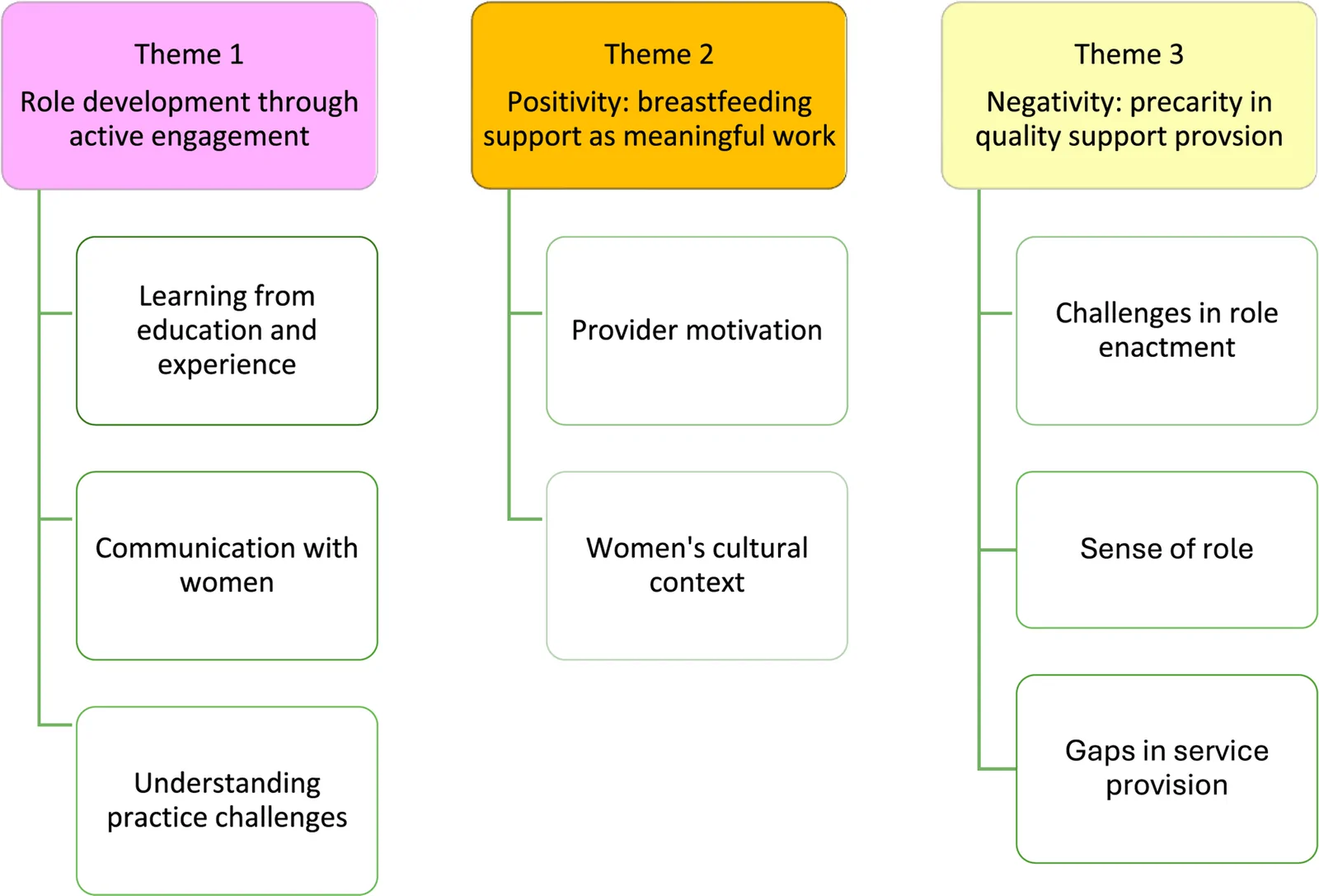
Thematic representation on factors underpinning breastfeeding support provision

The factors influencing support provision described by participants were positive and negative, and led to multilayered dynamic experiences of reward, joy, and sometimes disappointment. We developed three broad themes: *Role development through active engagement*,* Positivity: Breastfeeding support as meaningful work* and *Negativity: Breastfeeding support as precarious work.* A synthesis of findings was undertaken to identify influential factors in breastfeeding support provision. Themes and subthemes are presented in Fig. 1, and additional contributory data for themes are displayed in S3.

#### Role development through active engagement

This theme describes how providers developed their skills and confidence through education, experience and engagement with breastfeeding women, but experience and engagement were sometimes difficult to achieve in the low-breastfeeding context.

##### Learning from education and experience

Providers consistently reported that while UNICEF BFI breastfeeding education was a good foundation for effective breastfeeding support work, education programmes alone were insufficient to develop support skills. Ongoing experience and exposure to breastfeeding women were essential in learning to apply theory to practice. One participant explained:

*It’s alright doing the training and getting it all written down…but hands on…it’s the best thing. Learn as you go is the best thing that you’re gonna do’* [HCR12]

Peer supporters providing remote support engaged in self-directed learning through online group discussions with peers, attending conferences and reading relevant books. Participants in both role types were therefore proactive in accessing resources to develop support skills. Providers in healthcare roles also described informal learning strategies such as accessing the knowledge and guidance of experienced colleagues through observation and questioning, picking up “*wee tips*” [HCR08] when working with others. One participant reflected this continuous learning mindset by stating “*every day’s a school day*” [HCR06]. However, this depended upon ongoing exposure to breastfeeding, and active engagement with women was not guaranteed due to society’s low breastfeeding rates. This was a particular problem when trying to consolidate skills following breastfeeding education:

*Students … some of them would say they’ve seen virtually no breastfeeders*,* they’re coming out as a qualified midwife having not witnessed breastfeeding and then they are expected to support …that only comes through experience’* [HCR03]

Therefore, while UNICEF BFI education was valued, active engagement and in-person experience with breastfeeding women, and knowledge-sharing with experienced colleagues and peers, was consistently identified as key to learning support skills and developing in the role.

##### Communication with women

Across role-types participants emphasised the importance of understanding women’s intentions and emotions regarding breastfeeding in providing effective support. Establishing rapport and relationship facilitated provider’s ability to build women’s confidence to breastfeed, teach them practical skills and meaningfully praise the breastfeeding relationship. Providers working in settings that facilitated repeat contacts with women such as Sure Start settings, Continuity models and peer support via telephone were well positioned to establish relationships with women to build confidence as illustrated below:

*it’s just it’s like a light bulb going on, just the confidence, maybe that’s to do with the relationship she had with me. She knew me, she’s known me…and they believe in you*

Such settings allowed more relational continuity than the acute settings, and enabled providers to receive feedback on the effectiveness of their support. Thus, ongoing engagement with women not only fostered open communication and trust but also created opportunities for providers to receive feedback on the effectiveness of their support, which informed future practice.

##### Understanding practice challenges

Most providers in a healthcare role reported understanding management of breastfeeding challenges as key to learning appropriate support strategies to use with other women. Participants actively engaged with women to learn how to help them surmount difficulties without disrupting their breastfeeding trajectory. However, most participants in healthcare roles expressed concern that some colleagues gave inappropriate advice or ineffective support for manageable issues, despite their UNICEF BFI breastfeeding education. They were disappointed that others appeared to implement stop-gap measures such as supplementation of newborns rather than seeking breastfeeding-specific solutions to challenges. Inappropriate referrals to medical doctors for normal infant behaviour were also cited as examples of mismanagement, often leading to clinically unnecessary supplementation or intervention, as this provider explained:

*Because the baby is a bit unsettled it’ll be like “Oh they’ve got reflux I’ll call the GP and get you a prescription” and like these flippin’ medications have side effects* [NHCR10]

Participants viewed these decisions as stemming from limited understanding of typical infant behaviours due to a lack of exposure to breastfeeding in society, or an inability to address the ineffective breastfeeding practices which may underlie the babies’ behaviours. Several participants in a healthcare role also reported a reluctance amongst some colleagues to spend time engaging with women to assess foundational breastfeeding techniques and patterns. One reported a frequent sentiment: *“everybody wants a quick fix don’t they?”* [HCR06]. To illustrate this view of reluctance to engage with practice challenges in a timely manner, a specialist provider described receiving inappropriate referrals from UNICEF BFI trained colleagues for women establishing breastfeeding:

*My remit is meant to be complex in-depth breastfeeding challenges. That’s not what I’m seeing. I’m seeing simple latch issues over and over again* [HCR04]

The above quote illustrates the view amongst some participants across role-types that there were providers who spent limited time trying to understand normal breastfeeding practice and behaviours. Participants viewed this as a missed opportunity on two fronts, firstly, missed opportunities to effectively support women, and secondly missed opportunities to engage with challenges to further develop their own support skillset. Across all roles, many providers viewed that ongoing experience with breastfeeding support interactions, particularly those involving challenges, was a critical in learning how to support women well. Without active engagement, providers risked stagnating in their role, with less ability to offer women effective support.

#### Positivity: breastfeeding support as meaningful work

This theme described how within UNICEF BFI accredited settings, sustained motivation in the role was closely tied to providers relational continuity opportunities, meaningful engagement with women and positive collaboration with other support providers.

##### Provider motivation

Motivation to support breastfeeding women was represented across the dataset, stemming from personal and professional breastfeeding experiences, and reward in performing the support role. Strong motivation to support women was most represented in data from providers working in settings that allow for ongoing contacts. These included healthcare staff practising within Continuity of Carer models and Sure Start settings, and the peer supporters providing telephone support. These providers described feeling rewarded when witnessing women’s breastfeeding confidence and ability develop: *“You watch them*,* it’s like watching that tulip open and bloom”* [HCR03]. Positive feedback from women through repeated encounters was also a key motivator, with one participant noting that that their support can *“impact on somebody’s life”* [NHCR02]. Relational continuity therefore enabled providers to know that their guidance had made a positive impact, and reinforced satisfaction in the support role. In contrast, few participants in healthcare roles in acute settings where contact with women rarely spanned several episodes, described receiving positive feedback from women as a motivator to provide support. The lack of relational continuity meant there was little opportunity to see the outcome of the support provided. There was little evidence that providers had had any impact on women’s breastfeeding long-term, and these providers were less confident in the impact of their support: “*it would be nice sometimes when you put a lot of time and effort in to know if they succeeded” * [HCR09]

##### Women’s cultural context

Many providers viewed that their breastfeeding support was meaningful as they were contributing to societal change by normalising and valuing breastfeeding within a formula feeding culture. They were also supporting the woman’s family to understand and value breastfeeding, challenging the cultural norms where breastfeeding can be unfamiliar and undervalued:

*A lot of them [women] would be from young families locally to whom breastfeeding is pretty alien … one of the grandmothers… shaking her head and saying ‘That breastfeeding*,* it’s just not natural’…* [HCR10]

This finding was most strongly demonstrated by those providing support outside of acute healthcare settings. The peer supporters were particularly motivated by a strong belief that they could counter family infant feeding norms:

*I enjoy the first-time mothers who will go on to breastfeed all of their babies …they will support their sister… it’s the cascade of positivity that goes out from one person that has made it through* [NHCR03]

Thus, the broader breastfeeding advocacy work, even though ad hoc, of encouraging more understanding and acceptance of breastfeeding in women’s families helped these participants see their support work as meaningful and important. Supporting women over several episodes was required to see reward, in terms of building trust and offering tailored support and observing the outcomes of that support. Providers engaging with women and their families over time therefore led to a sense of contributing to a societal change where breastfeeding would become more valued.

#### Negativity: breastfeeding support as precarious work

Most providers across role types described a sense of precarity in quality support provision with missed opportunities for support, ineffective support and inconsistent role enactment. This theme highlighted that UNICEF BFI accreditation of services alone cannot always ensure high quality breastfeeding support provision when systemic constraints are ongoing in healthcare organisations, and breastfeeding is unfamiliar and undervalued in society.

##### Challenges in role enactment

Healthcare staff frequently reported barriers to fully enacting the support role as staff shortages, time constraints, and the sometimes low prioritisation of breastfeeding support in comparison to other care:

*There is a role for [breastfeeding support] to be recognised and valued as a clinical skill… there is a perception that we don’t have the time to sit and help women with the normal*,* the standard*,* the stuff that doesn’t seem very high tech… that first feed* [HCR04]

While a few providers in healthcare roles did report a temporary increase in time spent on breastfeeding support during periods leading up to UNICEF BFI re-accreditation, this effort was not sustained in everyday practice. Most participants acknowledged that despite healthcare system support for breastfeeding in principle, staffing shortages and time constraints in the postnatal period often hindered full enactment of the breastfeeding support role.

Some peer supporters also expressed concern about challenges in role enactment. For some, being restricted to telephone and text support lessened the quality of care they could offer. They were unable to apply skills learnt during their training such as assessment of positioning and attachment. A few peer supporters who wanted to contribute to in-person antenatal breastfeeding education alongside healthcare staff were disappointed when their offers were declined by healthcare staff. They felt this limited the positive potential of antenatal classes, and questioned whether there was some gatekeeping of the peer support role by healthcare staff:

*Why they wouldn’t just love for … a peer supporter to be in one of their antenatal classes*,* how are they not snapping that up? It might be in case somebody says anything rogue… or maybe it’s a bit too breastfeed-y or something… it’s probably a bit of defensiveness* [NHCR01]

Most of the peer supporters reported that although a telephone support service was worthwhile women needed more in-person breastfeeding support, and the peer support service was limited because they were often unable to apply their full skillset learnt in training. Thus, uniquely remote support was viewed by a few peer supporters as tokenistic. These participants felt that only providing remote peer support was a simple way to fulfil the requirements of UNICEF BFI accreditation without organising in-person peer support in clinical settings, leading to their disappointment:

*[remote peer support is] …lip service … just something that the Trust could put out there to tick a box* [NHCR10]

#### Sense of role

All participants, regardless of role type, viewed themselves as encouragers but not a *“pusher”* of breastfeeding [HCR07, NHCR07 and NHCR08] describing themselves as *“passionate*” about supporting women [HCR03, HCR05,NHCR05]. However, alongside the passion was a sense of being stretched in the role. Many expressed that they were *“picking up an awful lot of slack”* [NHR09] from healthcare colleagues who were perceived as disinterested or ineffective in breastfeeding support, despite having received UNICEF BFI training. As a result, these participants sometimes assumed additional work to ensure equal access to effective support for women. There was a common view that for some healthcare staff, breastfeeding was simply *“not their thing”* [HCR04]. Many peer supporters ascribed this to a lack of positive personal or professional breastfeeding experience which led to an undervaluing of and disinterest in breastfeeding, and subsequently limited engagement with the role, despite education:

*If you are not interested you don’t retain the information*,* and I know not every healthcare professional has an interest in supporting woman to breastfeed. If it’s not your thing then refer them* [NHCR03]

The peer supporters had varied perceptions of their role, shaped by their views on how well they felt their service was integrated within their individual healthcare Trusts, and their personal interactions with healthcare staff. Some reported receiving many referrals, and they felt recognised and appreciated by healthcare staff, describing positive relationships with midwives. Conversely, others felt that their service was undervalued, underused and potentially at risk of discontinuation, citing lack of referrals and little contact with the wider healthcare team. Two peer supporters describe this range of experience:

*I work with some community midwives who are really*,* totally passionate about breastfeeding but they have not got time and that’s when they’ll phone me and I feel so privileged* [NHCR03]

*I don’t hear from the [peer support] coordinator much at all. If the Trust doesn’t care about us*,* then why should we bother? … the peer support service will eventually fade away* [NHR09]

Therefore, participants reported that the sometime lack of interest in breastfeeding of other providers, a lack of collaboration and feeling isolated in the support role, lead to uncertainty about the consistency and sustainability of high-quality support services.

#### Gaps in service provision

Participants identified a gap between an ideal model of breastfeeding support characterised by knowledgeable, accessible face-to-face support, and the reality of service delivery where such support was often lacking, particularly in the early hours and days after birth. Providers working in acute settings noted that those in specialist breastfeeding roles were only available on weekdays, leaving a gap in skilled expertise on the wards during evenings and weekends. Several providers suggested introducing dedicated breastfeeding support staff to move between labour ward and the postnatal areas to support women and staff, to fill the gap. They recommended introducing a mid-level breastfeeding role, that is, a staff member who has additional breastfeeding education to midwives but less than lactation consultants. They also proposed facilitating in-person peer support as well as the remote service. However, a few providers in healthcare roles were reluctant to consider non-healthcare professionals providing in-person support, even when trained to do so. One midwife captured a concern:

*it’s a midwife’s job to do that. Why are we letting that go? No*,* no this [peer support] is a way to plug the gap until we really do what we need to do*,* which is to help midwives be passionate about it*,* and health visitors* [HCR04]

However, most providers in a healthcare role agreed with widening the pool of people offering breastfeeding knowledge, skill and enthusiasm, and welcomed multi-sector involvement:

*Breastfeeding it isn’t owned by midwives anymore*,* or health professionals*,* it’s much broader. As long as they are well trained*,* I think it’s great. I have changed in my view of that* [HCR07]

**Fig. 2:**
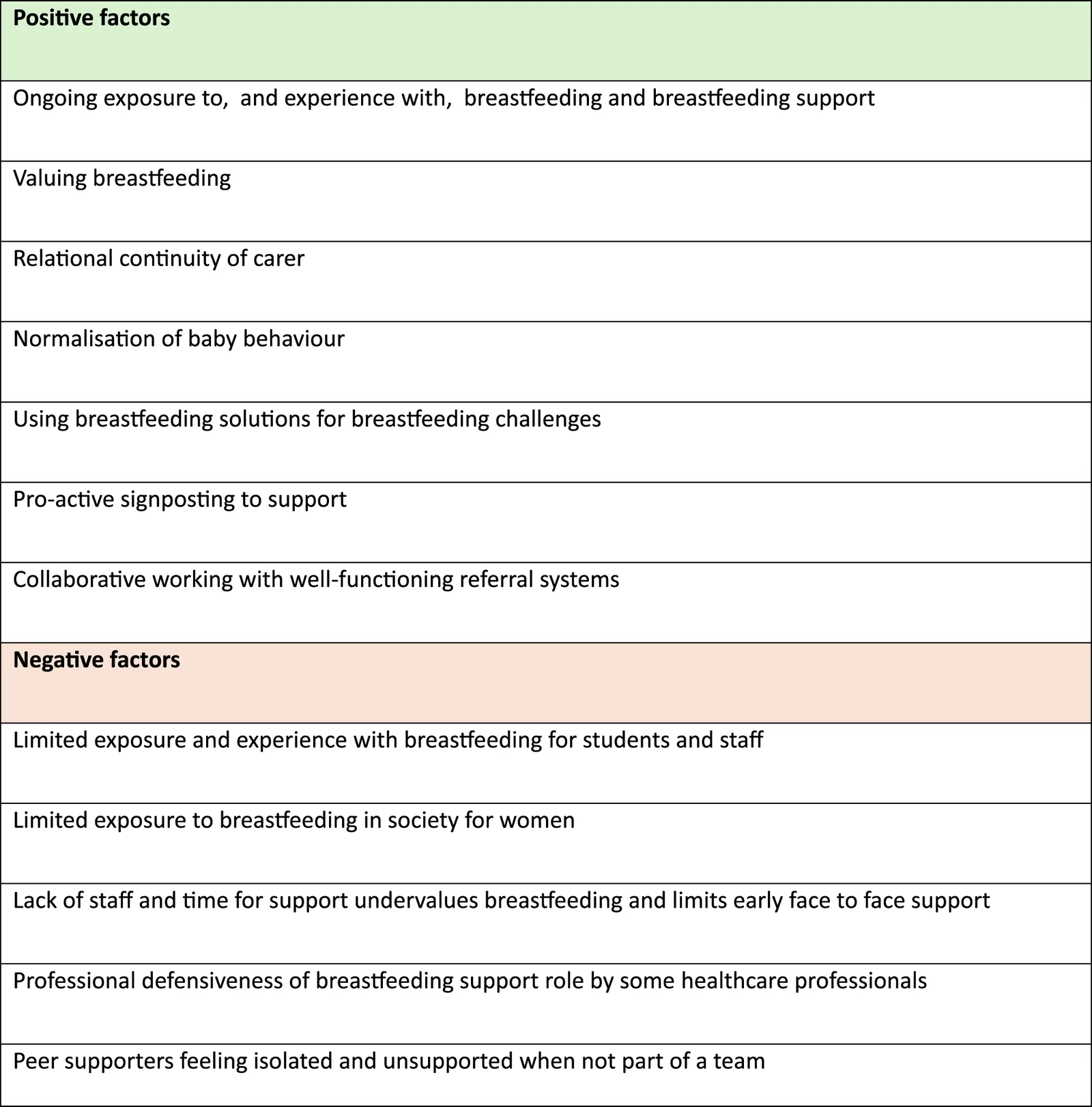
Synthesis of influential factors in support provision

This reflects the recognition amongst almost all participants that moving forward effective routine breastfeeding support requires contributions from community as well as healthcare sectors. Collectively, these findings have identified a range of positive and negative factors which influence support provision once UNICEF BFI accreditation is in place. These factors direct focus to what now must be addressed in improving breastfeeding support for women. See Fig. 2.

## Discussion

This study highlights the need for sustained, practice-based support to help providers build confidence, competence, and appreciation of breastfeeding, countering prevailing societal attitudes that may undervalue it. These findings suggest that theoretical breastfeeding education alone may be less successful than expected in societies with little practical understanding of breastfeeding. Findings suggest that when there is opportunity for providers to gain experience in breastfeeding support with knowledgeable others, relational continuity, positive multi-sector working, and a shared valuing of breastfeeding, providers are better enabled to fully implement the UNICEF BFI standards. Conversely, provider’s ability to offer effective support is limited when breastfeeding is not familiar to women, if breastfeeding is not consistently valued within broader healthcare settings despite BFI accreditation, or when breastfeeding is not familiar to, or valued by staff and women due to lack of exposure and experience. The importance of valuing breastfeeding as a relational and social practice, as well as a health behaviour, was highlighted. The concept of valuing breastfeeding extends beyond clinical knowledge of its health outcomes to recognising the importance of breastfeeding for mother-baby relationships and considering it worthy of time, resources and active support, including peer support. The extent to which breastfeeding is valued varied between individuals and settings due to influences such as personal beliefs and experiences, societal norms, and competing service demands. While the embedding of UNICEF UK Baby Friendly Initiative (BFI) standards such as Stage 2, which focuses on building an educated workforce [10] was reported positively, providers also highlighted concerns about a superficial approach to implementation of other standards. Those that are simpler and less resource-intensive tend to be prioritised and resourced, whereas standards requiring sustained investment such as adequate staffing and effective signposting to community parenting support (Stage 3, Standard 5) can be under-implemented. This reflects how breastfeeding is valued and underscores the need for consistent and equitable resourcing to ensure meaningful and comprehensive integration of all BFI standards.

Positive factors influencing breastfeeding support provision clustered around ongoing exposure to, and experience with breastfeeding, following BFI education. The importance of good quality education in preparing providers to support breastfeeding women has already been established in the literature [5, 6]. We found that after education, role modelling from experienced individuals can improve provider confidence and competence in supporting breastfeeding women, similar to earlier research [30–34]. These results suggest that in low-breastfeeding contexts, seeing knowledgeable others provide effective breastfeeding support is key to developing confidence and skill and sustaining motivation in the role. The finding that provider attitudes to breastfeeding and breastfeeding support are influenced by personal, professional and cultural contexts builds upon our earlier work [27] by suggesting that despite BFI education, a lack of understanding or valuing of breastfeeding can persist for some providers in low breastfeeding contexts. This result concurs with the evidence which recommends exposure to lived experience of breastfeeding alongside high quality education [6, 32, 34] and indicates that in low breastfeeding contexts there should be a mechanism for healthcare student exposure to positive breastfeeding relationships, and for staff, increased opportunities to learn from the breastfeeding support expertise of others in practice. An interesting finding was that relational continuity enabled consistent exposure to breastfeeding and opportunity to provide support, and it acted as a mechanism for feedback about the support provided. This offers new insight into the benefits of relational continuity in postnatal care for support providers, building on the existing literature that documents benefits for women [31]. These results indicate that in low breastfeeding contexts additional efforts should be made to support providers to maximise opportunities for repeat experience with breastfeeding women. In this study, relational continuity in community settings also afforded opportunity to engage with family members about breastfeeding, providing a new insight into the provider belief that their breastfeeding support could contribute to long-term societal change. Providers saw their role as shaping societal attitudes toward breastfeeding, with potential to impact future breastfeeding rates.

Australian research has demonstrated the importance of providers normalising common breastfeeding challenges [35] as many women lack personal and social reference points for typical breastfeeding behaviours [36, 37]. Our findings, from a setting with comparably lower breastfeeding rates, demonstrate that normalising usual breastfeeding patterns and practices, not only challenges, is central to the provider role. This insight adds to the evidence by showing that educating families how to appropriately interpret and respond to normal infant behaviours builds confidence and so may be particularly important in low breastfeeding contexts. Yet, the quality and consistency of information provided during breastfeeding support interactions is known to vary [38]. Conflicting information from healthcare professionals has been found to negatively impact breastfeeding outcomes including breastfeeding exclusivity and duration [39] as well as mothers’ breastfeeding experiences [40]. Our findings suggest that inappropriate and inconsistent advice continues to be experienced by women, and this can undermine their confidence to manage everyday breastfeeding challenges. Consequently, normal infant feeding behaviours may be interpreted as problematic, which can increase the likelihood of non-medically indicated supplementation with formula milk or earlier cessation of breastfeeding than originally planned [41]. This study therefore adds to an emerging issue in the literature, namely that misinterpretation of variants of normal infant behaviour [34–36, 42] risks medicalisation of infants and disruption of breastfeeding. Our finding highlighting such precarity in high quality support provision is somewhat surprising in the context of 100% UNICEF BFI accreditation of maternity and health visiting settings because healthcare professionals have received the relevant breastfeeding education. It is possible therefore that the low breastfeeding context could account for such inconsistent and conflicting advice because powerful cultural influences can influence infant feeding decisions [43] and the approach of support providers themselves [27]. So, while evidence from literature shows that ineffective support provision contributes to low breastfeeding rates [1, 6] our findings now suggest a circular relationship: low breastfeeding rates themselves negatively influence effective service provision due to limited provider exposure to breastfeeding support and normalisation of breastfeeding. It seems possible therefore that normalising the range of healthy infant behaviours within society could improve breastfeeding support practices, and subsequently, women’s confidence and breastfeeding rates. Yet, this first requires that support providers themselves become familiar with breastfeeding and value it through not only evidence-based theoretical education but also exposure to breastfeeding support and breastfeeding in practice, which may need a more concerted effort in low breastfeeding contexts. A structured approach to ensuring providers have meaningful opportunities to observe and engage with breastfeeding and breastfeeding support in practice is therefore required. Since experiential learning may not happen consistently in low-breastfeeding settings, healthcare organisations in such contexts should strive to ensure providers at all career stages can observe and engage with high quality breastfeeding support encounters. Such experiences may reinforce breastfeeding education, build confidence and sustain provider motivation in the role.

Another surprising finding was inconsistency in successful collaborative working. Although most of the professional providers reported a positive view of multisector working, there was evidence of some health professional gatekeeping of breastfeeding support opportunities for the providers not employed in healthcare settings. Positive multi-sector collaboration in breastfeeding support provision needs encouraged and monitored even within UNICEF BFI accredited settings. This findings contrasts with work by Byrom et al. [6] who identified effective teamwork and emotional engagement within UNICEF BFI-accredited settings. That study researched an established breastfeeding team, whereas the population in this study consisted of providers who were not part of recognised, formal breastfeeding teams at the time of data collection. Instead, there were ad hoc referral systems between the health professionals and lay supporters, with no shared physical or virtual space to build relationships across sectors and settings. Our findings instead align with earlier work reporting health professional defensive attitudes towards peer supporters which limited peer supporter access to women [44] and research demonstrating that a lack of robust supervision and support can lead to peer supporter demotivation [45]. Nonetheless, most participants in this study suggested mid-level breastfeeding roles within healthcare settings, and better integration of peer supporters into healthcare services, reflecting a desire to bridge this gap.

Surprisingly, some providers reported uncertainty about the sustainability of quality breastfeeding support services despite BFI accreditation of maternity and health visiting settings and the availability of third sector support providers. Resourcing adequate staff and time can indicate how breastfeeding support services are prioritised and valued within healthcare systems. The availability of specialist breastfeeding expertise only within office hours suggests that BFI accreditation does not always translate into full implementation of the BFI standards. In particular, the requirement for sufficient staffing to support instinctive, responsive breastfeeding behaviours [10] was often unmet in acute settings. Consistent with earlier work [44, 45] providers employed in healthcare roles in this study identified negative organisational and intra-role factors in breastfeeding support services which limited provider ability to consistently perform well. This was interesting given the BFI accreditation of these services. However, in this study there was also a reported increase in time spent on breastfeeding support during BFI re-accreditation periods, suggesting that healthcare systems can mobilise resources and attention to breastfeeding when needed. Consistently and systematically sustaining this focus is therefore required to demonstrate that breastfeeding is valued, and that breastfeeding support is always a valued skill.

### Limitations and future directions

Limitations included under-representation of postnatal ward and community midwives, likely due to COVID-19 factors such as restricted researcher access, staff redeployment, and increased workloads. Additionally, reliance on opt-in participation may have disproportionately represented positive attitudes toward breastfeeding support.

Future research should include the perspectives of postnatal and community midwives to reflect the entirety of the population who support breastfeeding women. Additionally, exploring the cultural and societal factors to be addressed for BFI to comprehensively embedded and sustained, would enable identification of strategies to normalise breastfeeding and baby behaviours in a low breastfeeding context. A study on women’s media health literacy relating to infant feeding and behaviours could contribute to countering societal misunderstanding and commercial interests that devalue and undermine confidence in breastfeeding.

## Conclusions

Breastfeeding support providers develop their confidence and skills through hands-on experiential learning and by working with colleagues who also value and promote breastfeeding. However, in low‑breastfeeding settings, limited exposure to breastfeeding and a lack of support for providers despite system‑level BFI accreditation, can undermine this development so providers can find it difficult to fulfil the breastfeeding support role. These findings demonstrate that what appears to work for providers is consistent exposure to breastfeeding dyads, opportunities for peer learning with supportive colleagues who understand breastfeeding practices, and strong team cohesion across professional and lay‑trained roles.

### Recommendations

When becoming Baby Friendly, a key recommendation is to design for relational continuity with women and for funded collaborative multi-sector working, from the outset. Sustainable, funded community-based multi-sector teams comprising opt-in healthcare staff with a strong commitment to breastfeeding and funded community advocates with lived experience could benefit both women and providers. Regular exposure to breastfeeding and repeated interactions with women would help with sharing knowledge and practice-based learning opportunities. Such teams would make breastfeeding expertise more visible in practice, provide role-modelling to students and new staff, and address the challenges such as limited exposure to breastfeeding, gaps in early postnatal support, and the sense of undervaluing and isolation experienced by some peer supporters. Team members should opt in to the team, based on their motivation and commitment to breastfeeding support. Integrating peer support more fully, and funding additional specialist breastfeeding support roles so that expertise is available beyond office hours, are recommended to improve support.

In addition, the process of learning to value breastfeeding should begin in childhood with a broad multi-sector strategy. Education incorporating visible positive breastfeeding relationships in schools could increase awareness of the value of breastfeeding, and normal baby behaviour at societal level. For healthcare students in low-breastfeeding contexts, particularly those in midwifery and health visiting, increased in-person experience is essential. Integrating mandatory practice hours at breastfeeding support groups and breastfeeding clinics into pre-registration curricula would ensure theoretical knowledge is coupled with meaningful real-world exposure. Investing in breastfeeding support as a clinical priority and societal need across health, community and education sectors, and investing in the development of those who provide it, can result in more sustainable and effective breastfeeding support provision.

## Supplementary Information

Below is the link to the electronic supplementary material.

Supplementary Material 1

Supplementary Material 2

Supplementary Material 3

## Data Availability

No datasets were generated or analysed during the current study.

## Abbreviations

GP: General Practitioner
HSCT: Health and Social Care Trust
IBCLC: International Board-Certified Lactation Consultant
PAG: Project Advisory Group
UNICEF BFI: United Nations’s Children’s Fund Baby Friendly Initiative
WHO: World Health Organisation
