# Supplementary material for: What works? A framework analysis of breastfeeding support provider perspectives in a low-breastfeeding region with baby friendly accreditation
Source: Int Breastfeed J. 2026 Aug 18;21:100. doi: 10.1186/s13006-026-00881-2 (PMC13760907; doi:10.1186/s13006-026-00881-2)
Supplement: Supplementary file 1 [file 13006_2026_881_MOESM1_ESM.docx]

S1 Eligibility Criteria

**Inclusion criteria**

•	Healthcare staff: Midwives, Maternity Support Workers, Health Visitors, Sure Start breastfeeding supporters and General Practitioners who have received breastfeeding training and who have experience of supporting women and infants to breastfeed

•	Trained breastfeeding support providers not employed in a healthcare role with experience of working with breastfeeding women and infants who have undertaken formal training from a course accredited by an external body such as a university, the UNICEF Baby Friendly Initiative, Open-College-Network and the Lactation Education Accreditation and Approval Review Committee (Lactation Education Accreditation and Approval Review Committee (LEAARC), or HSCT approved courses such as the Solihull Approach Peer breastfeeding Supporter Training.

•	International Board-Certified Lactation Consultant accredited breastfeeding supporters who have experience of working with breastfeeding women and infants

•	Over 18 years old

•	Ability to communicate in English

•	Ability to give informed consent

•	Currently engaging with breastfeeding women in Northern Ireland

•	Minimum 6 months experience of supporting women with breastfeeding

•	Any age range, gender, ethnicity, or socio-economic grouping

**Exclusion criteria**

•	Healthcare staff not engaging with breastfeeding women and healthy infants, for	example paediatricians and neonatal staff

•	Untrained breastfeeding supporters

•	Aged under 18 years

•	Inability to communicate in English

•	Inability to provide informed consent

•	Not currently engaging with breastfeeding women in Northern Ireland

•	Do not have at least 6 months experience of supporting breastfeeding women
