# Supplementary material for: What works? A framework analysis of breastfeeding support provider perspectives in a low-breastfeeding region with baby friendly accreditation
Source: Int Breastfeed J. 2026 Aug 18;21:100. doi: 10.1186/s13006-026-00881-2 (PMC13760907; doi:10.1186/s13006-026-00881-2)
Supplement: Supplementary file 2 [file 13006_2026_881_MOESM2_ESM.docx]

S2 Topic Guide

**Provider Perspectives Topic Guide**

**Background**

Aim:

To get participant talking comfortably, find out what led them to providing breastfeeding support and their work/volunteer role.

**Perception of Role**

Aim:

To find out how participants view their role, how they view their influence in helping women overcome common challenges, whether they are aware of/ make use of other types of breastfeeding supporter(s) when they cannot solve challenges, any issues around teamwork/ territoriality, whether they believe they can help a woman surmount difficulties, and whether they value WHO guidance and believe it to be achievable**.**

**Views on training**

Aim:

To find out perceptions of what is useful/ not useful in breastfeeding training and why, does it prepare them for the reality of the role, any other influencing factors on knowledge and skills (experience/networking), identify any training needs and find out about use/ lack of use of existing tools/ evidence-base and why.

**Motivators and Deterrents**

Aim:

To identify motivating or deterring factors in providing breastfeeding support, how they measure their successes, and whether they receive any support to carry out their role.

**Personal views about breastfeeding**

Aim:

To explore any personal conflict with expected professional/ volunteer values/views, to explore how the participants value breastfeeding.

**Current state of affairs**

Aim:

To find out participants’ perceptions of why Northern Irish breastfeeding rates are so low, views on current breastfeeding support provision, what has changed during the pandemic, whether new formats of breastfeeding support have replaced face-to-face formats and how participants feel about this.

**Close**

Aim:

To explore how participants see their future in breastfeeding support provision, thoughts on an “ideal” model of breastfeeding support in Northern Ireland, and to close on a less intense topic.
