# Supplementary material for: What works? A framework analysis of breastfeeding support provider perspectives in a low-breastfeeding region with baby friendly accreditation
Source: Int Breastfeed J. 2026 Aug 18;21:100. doi: 10.1186/s13006-026-00881-2 (PMC13760907; doi:10.1186/s13006-026-00881-2)
Supplement: Supplementary file 3 [file 13006_2026_881_MOESM3_ESM.docx]

S3: Examples of additional contributory data for themes

| **Theme & Description** | **Sub themes** | **Quote** |
| --- | --- | --- |
| ***Role development through active engagement*** | *Learning from education and experience* | *I watched a more experienced midwife. You know when the babies have a preference for a side? She was just saying bring them in [to the mother’s body] as if they're going to that [preferred] side... I was like I'm 10 years out and I never knew to do that [HCR06]*  *You learn from each other as well and you see other midwives working, that would be a big part of it [HCR09]* |
|  | *Communication with women* | *It was about her confidence in herself to produce enough milk…I’m saying to her “Gosh how brilliant are you! I mean there’s no bottlefeeder would be gaining weight like that!” … it is about confidence [HCR07]* |
|  | *Understanding practice challenges* | *There is a massive over-medicalisation of babies [HCR02]*  *Because of a lack of understanding, a lack of clinical skill and experience, and the lack of passion for supporting Mummies with putting in place the basics well … you can just see the cascade happening of ..its reflux, it's cow’s milk protein intolerance, it's tongue tie. If you can put a label on it, then it's not the Mum's fault, it's not the staff’s fault, it's not something that you're doing or not doing it's just happening by chance.. but it's really not [HCR04]* |
| ***Positivity: Breastfeeding support as meaningful work*** | *Provider motivation* | *Just having a Mum come back and say, you know “that's made such a difference and I wouldn't be breastfeeding without your help” [NHCR11]*  How does that make you feel? [Interviewer]  *(NHCR11 brings hand to chest) “Sorry” (reddens and eyes brim with tears) “really good.” [NHCR11]*  *I was sitting there just thinking this feels amazing. Every woman should get to feel this, I want to help women feel this [NonHCR02]* |
|  | *Women's cultural context* | *We have been a very formula feeding society, when a Mum hits those problems her extended family around her talk about what they know and they say things like “Are you sure he's getting enough milk? If he’s hungry again maybe you should give him a bottle. I could help give a bottle.” It's all coming from a good place, but there's just not a lot of understanding of what is normal, breastfeeding baby behaviour here [NHCR02]*  *“so proud…up against it all, first breastfeeder in the family…she is an utter pioneer…her sisters are looking at her and thinking Wow!” [HCR10]* |
| ***Negativity: Breastfeeding support as precarious work*** | *Challenges in role enactment* | *Mums probably perceived from me like a state of rush rush rush rush it’s just awful. Do you know they probably felt they couldn't say actually, could you help me? [HCR06]*  *The Trust peer support training was more kind of positioning and attachment … that’s your bread and butter but you're not seeing the Mummies in person, it's sometimes hard to really help them a lot with positioning and attachment [NHCR06]* |
|  | *Sense of role* | *It depends how busy your unit is. I do think it's valued, I think, especially when the Trusts are trying to go to get Gold there is definitely more of an emphasis on breastfeeding support [HCR05]*  *“I think to be honest that one has to have an interest in it and that interest could be influenced by personal influence…it's there in the training and it's part of the job but in all my time, I've met midwives who are very passionate and midwives who are not a bit passionate at all, you know, they're not [HCR03]*  *It just depends on who knows you and who trusts you. There was one infant feeding lead, and she and I worked so closely together it was actually amazing and brilliant. And then a different infant feeding lead and I just, you know ... she doesn't value my knowledge and experience in the same way as the other one did [NHCR03]*  *It was lip service for peer support for breast feeding.. you give so much of your time and your energy to supporting these women and it really felt like it wasn't appreciated or acknowledged, or you know, it was just something that the Trust could put out there to tick a box [NonHCR10]* |
|  | *Gaps in service provision* | *There's a big gap there between peer support and health care professionals that are like the breastfeeding counsellor role… you don't need to speak to an IBCLC ninety percent of the time… that inbetween stage is definitely missing where people are more educated than peer support but not IBCLC” [NHCR03]*  *Someone like an expert on a shift … give you a hand if you're really struggling to think of anything else to try to get that baby on, or even just for advice on how long you could leave it sometimes [HCR09]* |
